# Exploration of lung mycobiome in the patients with non-small-cell lung cancer

**DOI:** 10.1186/s12866-023-02790-4

**Published:** 2023-03-25

**Authors:** Yiming Zhao, Junqi Yi, Juanjuan Xiang, Wei Jia, Anqi Chen, Liyu Chen, Leliang Zheng, Wen Zhou, Minghua Wu, Zheng Yu, Jingqun Tang

**Affiliations:** 1 Department of Thoracic Surgery, the Second Xiangya Hospital, Central South University, Changsha, Hunan China; 2 Department of Microbiology, School of Basic Medical Science, Central South University, Changsha, Hunan China; 3 Hunan Key Laboratory of Early Diagnosis and Precise Treatment of Lung Cancer, Changsha, Hunan China; 4 NHC Key Laboratory of Carcinogenesis and the Key Laboratory of Carcinogenesis and Cancer Invasion of the Chinese Ministry of Education, Cancer Research Institute, School of Basic Medical Science, Central South University, Changsha, Hunan China; 5 School of Chinese Medicine, Hong Kong Baptist University, Kowloon Tong, China

**Keywords:** Non-small-cell lung cancer, Mycobiome, *Alternaria arborescens*

## Abstract

As the Human Microbiome Project (HMP) progresses, the relationship between microbes and human health has been receiving increasing attention. A growing number of reports support the correlation between cancer and microbes. However, most studies have focused on bacteria, rather than fungal communities. In this study, we studied the alteration in lung mycobiome in patients with non-small-cell lung cancer (NSCLC) using metagenomic sequencing and qPCR. The higher fungal diversity and more complex network were observed in the patients with NSCLC. In addition, *Alternaria arborescens* was found as the most relevant fungus to NSCLC, and the enrichment of it in cancerous tissue was also detected. This study proposes that the changes in fungal communities may be closely related to lung cancer, and provides insights into further exploration the relationship between lung cancer and fungi.

**Supplementary Information:**

The online version contains supplementary material available at 10.1186/s12866-023-02790-4

## Introduction

Microbes have been implicated in human health [1], and some researches have reported that microbes are also related to lung cancer [2–6]. The microbial composition of the lower airways of lung cancer patients was unique, and this microbial alteration is considered associated with lung carcinogenesis [7, 8]. A dysregulated microbiota has a role in propagating and maintaining a chronic inflammatory environment [9, 10]. Some lung microbes affect T helper 17 cells which were key in modulation of lung immune status in health and disease [11]. In addition, microbes can directly affect protumorigenic pathways in epithelial cells [7].

Although the opinion that the lungs are sterile has been abandoned, researches on lung microbes have mainly focused on bacteria [12, 13]. Fungi are often neglected due to their low content. However, the mycobiome can play a beneficial or pathogenic role [14]. Fungal genera that have been detected in the pulmonary mycobiota mainly include *Candida*, *Malassezia*, *Neosartorya*, *Saccharomyces*, and *Aspergillus*. A systematic review showed that the human mycobiome, along with its interactions with the human bacteriome and the host, is implicated in the promotion and progression of carcinogenesis [15]. *Candida albicans* exhibit an oncogenic potential in oral cavity cancer [16–19]. *Malassezia* has been found among colorectal carcinoma patients, whilst an increased number of *Basidiomycota* have been suggested to be related to more advanced stages of this kind of cancer [20–22]. However, the relationship between lung mycobiome and lung cancer remains unclear. In this study, we characterized the alteration of fungal lung communities in patients with NSCLC and the Non-NSCLC groups, and found *A*. *arborescens* were enriched in NSCLC tissues.

## Methods

### Study population and sample collection

To study the lung mycobiome in patients with NSCLC and to verify the enrichment of key fungi in cancer tissues, 66 patients were enrolled with following inclusion criteria: (1) 40 < Age < 70; (2) No antibiotics within three months; (3) No immunosuppressive drugs within six months. All individuals included in this study had no evidence of infection, sepsis or active tuberculosis. The research presented here has been performed in accordance with the Declaration of Helsinki, and cases enrolled in this study were collected and approved by the ethical review committees from the second Xiangya hospital, Central South University, China (Ethical approval number: 2019–155). The patients were informed about the sample collection and had signed informed consent forms.

As for metagenomic sequencing and estimation of lung fungal content, the bronchoalveolar lavage (BAL) was collected from 38 individuals, including 24 newly diagnosed NSCLC and 14 Non-NSCLC patients (Table 1). There was no significant difference between the NSCLC and Non-NSCLC in terms of age, smoking status, and gender. To better quantify the cancer progress, the patients were divided into 10 groups according to TNM stages (Table 2). The BAL samples were obtained under sterile conditions by instillation and aspiration of 20 ml of 0.9% NaCl from the bronchoscope. The samples were frozen in sterile containers and stored at − 80 °C. The same amount of NaCl liquid was also collected as for blank control.

**Table 1:** Clinical Characteristics of patients for metagenomic sequence

Feature	NSCLC patients		Non-NSCLC Patients	
**Patients(*n* = 38)**	24(63.2%)		14(36.8%)	
**Genders(*n* = 38) ***p* = 0.929	Male	12	Male	8
	Female	12	Female	6
**Age(*n* = 38) ***p* = 0.125	≤ 50	9	≤ 50	7
	> 50	15	> 50	7
**Smoking status(*n* = 38) ***p* = 1.000	Smoker	4	Smoker	2
	Non-smoker	20	Non-smoker	12
**TNM staging(*n* = 22)**	T1aN0M0	2		
	T1bN0M0 T1cN0M0 T2aN0M0 T2bN1M0 T3N1M0 T4N0M0 Tia	7 1 8 1 1 1 1		

All patients are immunocompetent in the cohort

**Table 2:** Cancer stage

Staging and Grading Cancer	Quantitative digital
Non-NSCLC	0
Tia	1
T1aN0M0	2
T1bN0M0	3
T1cN0M0	4
T2aN0M0	5
T2bN0M0	6
T2cN0M0	7
T3aN0M0	8
T3bN0M0, T2bN1M0	9
T3cN0M0, T3N1M0, T4N0M0	10

The cancer tissues and matched para-cancerous tissues from additional 28 patients with NSCLC were included for determination of abundance of *A. arborescens*. The cancerous and para-cancerous tissues were separated by surgical scissors and put in the sterile lyophilization tubes. PBS was used as the control. All samples in the study were collected in sterile conditions. The details of clinical data are presented in Table 3.

**Table 3:** Clinical Characteristics of patients for testing *A. arborescens* enrichment

Feature	NSCLC patients	
**Genders**	Male	15
	Female	13
**Age**	≤ 50	13
	> 50	15
**Smoking status**	Smoker	5
	Non-smoker	23
**TNM staging(*n* = 28)**	Tis	7
	T1aN0M0	1
	T1bN0M0 T1cN0M0 T2aN0M0 T3N0M0 T4N0M0 T4N2M0 T4N1M1a T1cN2M0 T1bN0M1a	4 3 7 1 1 1 1 1 1

All patients are immunocompetent in the cohort

### Metagenomic sequence

The DNA was isolated from samples, and Nanodrop (2000/2000c, American) was used to quantify the DNA. The DNA with 1.8 < A260/A280 < 2.0 and concentration > 20 ng/uL was used for following metagenomics sequencing. The blank controls and isolation kits were used as negative controls to quantify the DNA, but they didn’t pass the threshold for sequencing. The metagenomic sequencing was performed using the paired-end sequencing method on the Illumina platform (San Diego, CA, USA). The DNA was sheared by ultrasonication (Covaries, Woburn, MA). The sheared DNA fragments were end-repaired (DNA End Repair Mix) at 20 °C for 30 min. The DNA fragments were purified by QIAquick PCR Purification Kit (Qiagen) and A-tailed using A-Tailing Mix. Libraries were checked using Bioanalyzer 2100 (Agilent) and quantified using the ABI StepOnePlus Real-Time PCR System. Libraries were sequenced on an Illumina platform.

### Estimation of lung fungal content

To estimate the lung fungal content, a qPCR approach based on ‘FungiQuant’ was used [23]. The FungiQuant primers were FungiQuant-F: 5’-GGRAAACTCACCAGGTCCAG-3’ and FungiQuant-R: 5’-GSWCTATCCCCAKCACGA-3’ yielding products of approximately 350 bp in the fungi 18S rRNA gene. *E. penicillium* DNA extracted from DNeasy UltraClean Microbial Kit (12,224 Qiamgen, Germany) was used to establish a standard curve in tenfold serial dilutions with 10 ng – 1 pg in each run. BAL samples were used to extract total fungal DNAs (QIAamp UCP Pathogen Mini Kit 50,214, Qiagen, Germany). The optimized conditions included the reaction mixture (20 μl) for qPCR contained ChamQ Universal SYBR Color qPCR Master Mix (Vazyme Biotech, Jiangsu, China), forward and reverse primer (final concentration 400 nM), the templet DNA and molecular-grade water, with all reactions performed in triplicates on the 7900HT Real Time PCR System (Applied Biosystems). We used the following PCR conditions: 3 min at 50 °C for UNG treatment, 10 min at 95 °C for Taq activation, 15 s at 95 °C for denaturation, and 1 min at 65 °C for annealing and extension × 50 cycles. Fungal content of each BAL sample was calculated according to the standard curve.

### Fungal taxonomic profile

Raw sequences were processed to remove low-quality sequences using fastp [24] (Version 0.21.0). Fastuniq [25] (Version 1.1.0) was used to remove duplicates in paired short DNA sequence reads in a FASTQ format. Human sequences were filtered out using the human reference genome (hg37) by bowtie2 [26] (Version 2.3.5).

The remaining high-quality reads were used for taxonomic classification by Kraken2 [27] (Version 2.0.7). We used ‘kraken-build’ tools to build the fungi-kraken2 database (kraken2-build –download-library fungi), and all fungal nucleotide sequences from NCBI (www.ncbi.nlm.nih.gov) were included. Next, the Bracken was used to obtain the read count for different fungi to estimate relative abundance. The read counts table of several levels (e.g., phylum, class, order, family, genus, species) were rarefied to the minimum fungal read counts to reduce the effects of uneven sampling in the cohort by using R package ‘picante’ [28] (Version 1.8.2). After the above processing of the raw sequences, we obtained 41,728 reads in each sample, which were annotated to a total of 341 fungal species.

### Fungal diversity in the cohort and rarefaction curve analysis

The Shannon–Wiener index and Gini-Simpson index were calculated to determine the fungal alpha diversity. In addition, the Bray–Curtis dissimilarity indices between samples at the species level were calculated to estimate beta diversity by using R package ‘vegan’ [29]. The permutational multivariate analysis of variance (PERMANOVA) and principal co-ordinates analysis (PCoA) were performed to estimate the between sample (β) diversity. Rarefaction analysis was performed to assess the fungi richness in the NSCLC patients and non-NSCLC. For a given number of samples, we performed random sampling 1000 reads in the cohort with replacement and estimated the total number of species that could be identified from these samples by the richness estimator.

### Co-occurrence network construction and analysis

To explore the different fungal correlations between NSCLC and Non-NSCLC groups, the co-occurrence networks were constructed based on the relative abundances of different fungi at the species level by using FastSpar [30] (Version 1.0). Only robust (*r* > 0.8 or *r* <  − 0.8) and statistically significant (*p* < 0.05) correlations were incorporated into network analysis. Network visualization in Fig. 2 and network parameters (i.e., degree, betweenness, diameter, and cluster coefficient) analysis were made with Gephi (version 0.9.2) using the undirected network (where edges have no direction) and the ‘Fruchterman-Reingold’ layout. Afterwards, we used UMAP algorithm in python ‘umap’ library to display all data into one plane. The two networks were parsed into modules in the ‘igraph’ R package (Version 1.2.6). After modularizing, the networks were re-visualized according to module attributes and the two sub-networks were extracted by using Cytoscape (Version 3.8).

### Regression model construction

In order to study the relationship between various fungi with the development of NSCLC, we used two regression models in machine learning. The Ordinary Least Square (OLS) model by the function ‘lm’ in R was used to estimate adjusted R^2^ based on cancer stage and relative abundance for each fungus. To investigate the effect of the inclusion of multiple independent variables (fungi) on the regression model, we first reduced the different multiple variables to two variables using the PCA algorithm. The results show that two-dimensional data produced by PCA can explain more than 99.95% of the explainable variance. Next, we introduced a ridge regression model to eliminate the effect of covariance between multiple variables on the model. The PCA and ridge regression was used by python ‘sklearn’ library.

### Key fungi detection between NSCLC and Non-NSCLC groups

The rarefied read counts of fungal taxa at species level were used to achieve Random forests by the R package ‘rfPermute’ (Version 2.1.81), and different species were identified by R package ‘DESeq2’ (Version 1.26.0) between NSCLC and Non-NSCLC groups.

### Test for *A. arborescens* enrichment

To test enrichment of *A. arborescens*, we used the nested PCR on 28 newly diagnosed patients with NSCLC. Nested PCR involves two sequential amplification reactions, each of which uses a different pair of primers. The first amplification primers are the paired primers were the same as the primers (FungiQuant) for estimating fungal content above. The product of the first amplification reaction is used as the template for the second PCR, which is primed by oligonucleotides that are placed internal to the first primer pair. And the second primers are specific for *A. arborescens* primers which were designed using Primer blast for amplification. Meanwhile, all primers were validated using gradient qPCR to detect the annealing temperature and the specificity of primers. Primers were obtained from Sangon Biotech (Shanghai, China). The primers for *A. arborescens* were AA-F: 5’- CAAATATGAAGGCGGGCTGGA-3’, AA-R: 5’-TGTCCTAGTGGTGGGCGAAC-3’.

Appropriate tissue samples including paired cancer tissue and adjacent cancer tissue were used to extract total fungal DNAs (Qiagen Blood & Tissue Kit, GER). At the same time, an equal volume of PBS DNA was extracted as the negative control and the DNA of *Malassezia globosa* was extracted as the positive control. The first round of amplification was performed in a 50 μl reaction mix containing 25 μl Premix Taq DNA polymerase (Takara, Dalian, China), 1.2uM for each primer and 5ul of template DNA. To minimize air-borne contamination, all steps were performed in a class 2 laminar flow safety cabinet. The temperature profile for amplification was as follows: initial denaturation at 94 °C for 4 min, denaturation at 94 °C for 30 s, annealing at 55 °C for 30 s, and extension at 72 °C for 1 min, for 35 cycles, followed by a final extension at 72 °C for 10 min. Two percent AGAR gel electrophoresis was used (120v 35 min) and the FungiQuant DNA fragments were cut under the UV light and extracted through QIAquick Gel Extraction Kit (Qiagen, GER), whose construction was detected by Nanodrop. In the step of PCR, an equal amount of DEPC treated water was also used to replace the template DNA, which was used to exclude the contamination caused by the PCR experimental system. The reaction mixture (20 μl) for PCR contained ChamQ Universal SYBR Color PCR Master Mix (Vazyme Biotech, Jiangsu, China), forward and reverse primer (final concentration 400 nM), and the extracted FungiQuant DNA fragments (5 ng). The Vazymecycling program was 40 cycles and consisted of 95 °C for 10 s and 56 °C for 30s and 72 °C for 1 min with an initial cycle of 50 °C for 2 min and 95 °C for 2 min. All steps in nested PCR were described in Additional file 1: Fig. S1. In addition, to test for contamination in the test for *A. arborescens* enrichment operation, a negative control (possible contamination in the PCR and environment) and a positive control were set up in the pre-amplification step and examined by using agarose gel electrophoresis. In the nested PCR process, a negative control was also set up (containing possible contamination during the sample preservation, pre-amplification and PCR process), and all results showed that there was no contamination that could affect the results during the test (Additional file 1: Fig. S2).

Assuming that for all templates and primers a cycle equally doubles the number of template DNA, the relative abundance of a certain strain (i) can be calculated as follows:

$$\mathrm{Relative\ abundance }(\mathrm{i}) =\frac{{(\frac{1}{2})}^{C{T}_{i}}}{{(\frac{1}{2})}^{C{T}_{c}}}={(\frac{1}{2})}^{C{T}_{i}-C{T}_{c}}={(\frac{1}{2})}^{\Delta CT}$$

The cycle threshold of strain i primer and common primer (total fungus) are represented by CTi and CTc, while ΔCT denotes the difference between them. From the equation, the logarithm of the relative abundance negatively correlates linearly with ΔCT.

### Other statistical analysis and data visualization

For all statistical analysis and prediction models, python 3.8.5 and R 3.6.1 were used. The baseline data in Table 1 were obtained by using R package ‘compareGroups’. The data visualization process in this article were implemented in python library ‘matplotlib’ or R package ‘ggplot2’.

## Results

### Greater fungal diversity in patients with NSCLC

**Fig. 1:**
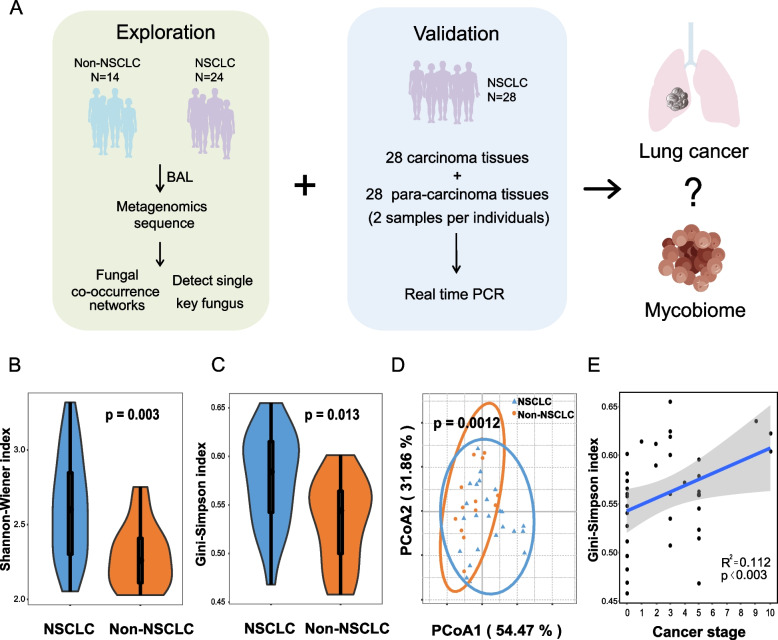
Characterization of lung mycobiome. (**A**) The scheme of the total research design. The *α* diversity between NSCLC and Non-NSCLC described using Shannon–Wiener index (**B**) and Gini-Simpson index (**C**), at species level respectively (Wilcoxon test). (**D**) PCoA based on the Bray–Curtis dissimilarity index shows the β diversity, in which the blue circles and orange triangles represent NSCLC and Non-NSCLC, respectively (PERMANOVA, *p* = 0.0012). (**E**) The relationship between Gini-Simpson index in different samples and cancer stage, which is calculated by the least-squares linear regressions, with 95% confidence intervals (gray-shaded areas)

To explore the mycobiome difference between NSCLC and non-NSCLC groups, we performed metagenomic sequencing in BAL from these two groups (Fig. 1A). The fungal composition in the cohort was dominated by the species *Lasiodiplodia theobromae* and *Malassezia globosa*, representing 64.62% and 11.83% of the fungi, respectively, followed by G*rosmannia clavigera* (4.7%), *Botrytis fragariae* (2.8%) and *Aspergillus flavus* (2.3%). Thus, about 85% of fungi were covered by the top five most abundant species in both Non-NSCLC and NSCLC patients. Among these five species, *Botrytis fragariae* was more abundant in patients with NSCLC than Non-NSCLC (Additional file 1: Fig. S3A). The species rarefaction curve for each sample was performed to approach saturation, indicating that the sequencing depth was adequate and samples in NSCLC exhibited higher richness (Additional file 1: Fig. S3B). Indeed, the higher fungal α diversity in patients with NSCLC was confirmed by the Shannon–Wiener index and Gini-Simpson index (*p* = 0.013) (Fig. 1B-C). The complex compositions of fungi were visualized on a two-dimensional plane using PCoA analysis and PERMANOVA test, demonstrating the significantly different fungal community composition between the two groups (*p* = 0.0012) (Fig. 1D). The *α* diversity elevated with the progress of NSCLC (Fig. 1E). Furthermore, the fungal communities in patients with NSCLC showed a significantly higher β-diversity (*p* = 0.003) (Additional file 1: Fig. S3C). The qPCR was also performed to check the fungal content in the lung, and it showed increased fungal content in patients with NSCLC (Additional file 1: Fig. S3D).

### More complex co-occurrence fungi network in patients with NSCLC

**Fig. 2:**
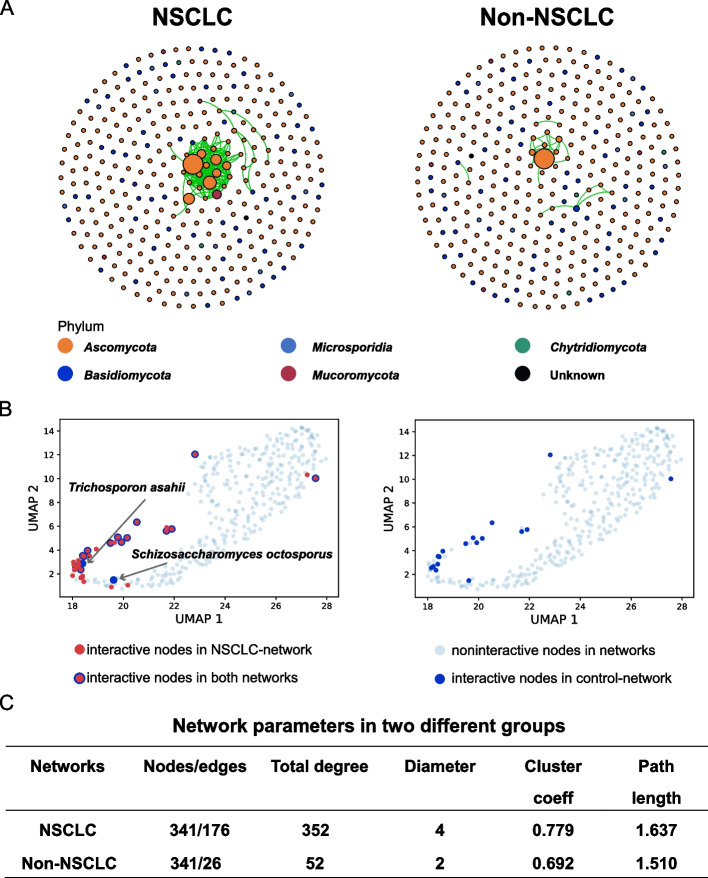
Networks of co-occurrence fungi across two groups. (**A**) The relative abundance of each fungi is used to construct the co-occurrence networks by sparcc method. Only the nodes with correlation coefficient (*r* > 0.8 or *r* <  − 0.8 significant at *p* < 0.05) are considered. The nodes are colored according to phylum. Green edges represent positive correlations and red edges represent negative correlations. Node size is proportional to the betweenness centrality of each species, and edge thickness is proportional to the weight of each correlation. (**B**) UMAP analysis shows each node status from NSCLC to Non-NSCLC. The two plots show different nodes in NSCLC and the Non-NSCLC networks from left to right, which paired to Fig. 2A. Interactive node means the node of which degree is not zero. (**C**) The network parameter is calculated in two networks. Total degree is the sum of edges on each node, representing the number of other nodes (species) in the network which are connected with the given node. Diameter is the largest distance between two nodes in a network. Clustering coefficient shows the extent by which a node is connected to its neighbors. Path length represents the nearest distance between two nodes

To study the community differences of fungi between the two groups, we constructed two co-occurrence networks of NSCLC and non-NSCLC groups. The degree distributions of the fungal co-occurrence networks conformed to the power-law distribution, indicating that the fungal community was constructed in a non-random way (Additional file 1: Fig. S4). The pattern of co-occurrence was more obvious in the NSCLC network, and the majority of taxa belonged to *Ascomycota* phylum in both networks (Fig. 2A).

To better observe the distribution of fungi in the co-occurrence network in the overall sample, the UMAP algorithm was used to downscale the relative abundance data. All the fungi are displayed in a two-dimensional plane in Fig. 2B. Some kinds of fungi marked in red points represented the exclusive taxa in the NSCLC co-occurrence network, which concentrated in the lower left corner of the plane after downscaling. Meanwhile, two kinds of fungi marked in blue points presented the exclusive taxa in the Non-NSCLC network. Compared to the Non-NSCLC group, fungal communities in the NSCLC group had a more complex network with higher edges (*n* = 176), a higher total degree (*n* = 352), and a higher clustering coefficient (0.779, Fig. 2C).

### Fungal community structure associated with development of NSCLC

**Fig. 3:**
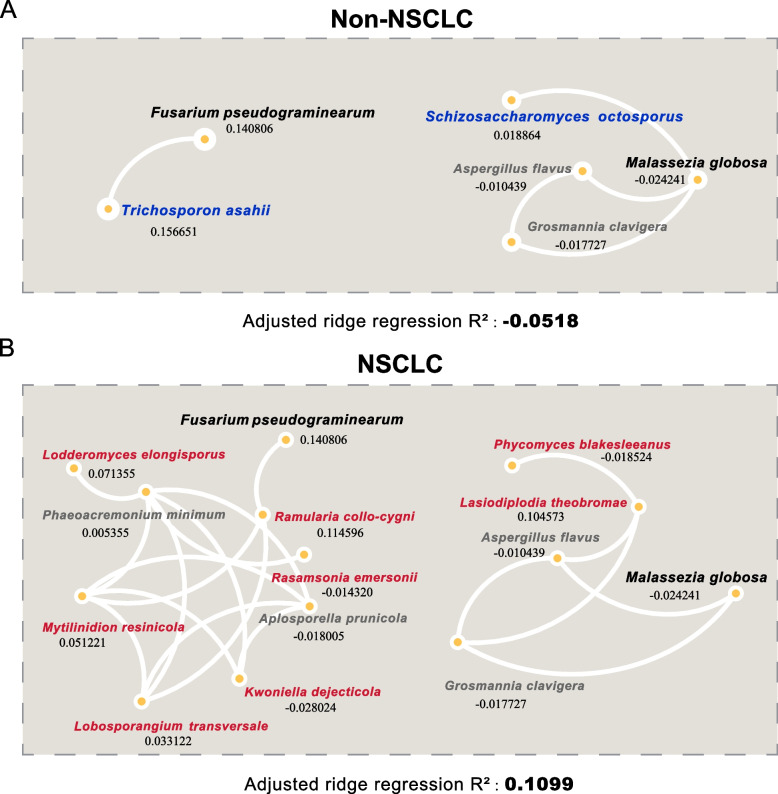
Four sub-networks extracted from Non-NSCLC co-occurrence network and NSCLC co-occurrence network. (**A**) Two sub-networks extracted from Non-NSCLC network, which contain two interactive nodes (blue font) only occurred in Non-NSCLC network but not in NSCLC network. (**B**) Two sub-networks extracted from NSCLC network, which pair with the two networks in Fig. 3A. In the Fig. 3, the nodes with red font are the interactive nodes only occurring in NSCLC network and the nodes with black bold font are first neighbor connected with blue font nodes. The grey font nodes represent interactive nodes in both networks. And the numbers under each node labels are the adjusted R^2^ for every species in Ordinary Least Square (OLS) model

We tracked two kinds of fungi (*Trichosporon asahii* and *Schizosaccharomyces octosporus*) that were isolated (degree was zero) in the co-occurrence networks after the transition of pattern from the Non-NSCLC to NSCLC, and extracted the sub-networks of them (Fig. 3A-B). In the two sub-networks, *Fusarium pseudograminearum and Malassezia globsa* were as interactive nodes connecting other fungi. With the disruption of co-occurrence relationship between *T. asahii* and *F. pseudograminearum*, more exclusive fungi in the NSCLC co-occurrence network uncovered. Furthermore, with the disruption of co-occurrence relationship between *S. octosporus* and *M. globosa*, this situation occurred equally. To explore the effect of fungal community alteration on the development of NSCLC, we constructed a model through ridge regression, which suggested that altered fungal community had a greater potential for the progression of NSCLC (adjusted R^2^ from -0.0518 to 0.1099). Notably, the contribution to the development of NSCLC (adjusted R^2^) for each fungus did not increase with the degree which is the size of edges for them (Additional file 1: Fig. S5A). It suggested that the fungi isolated in the networks cannot be ignored either.

### *Alternaria arborescens* as key fungi related to NSCLC

**Fig. 4:**
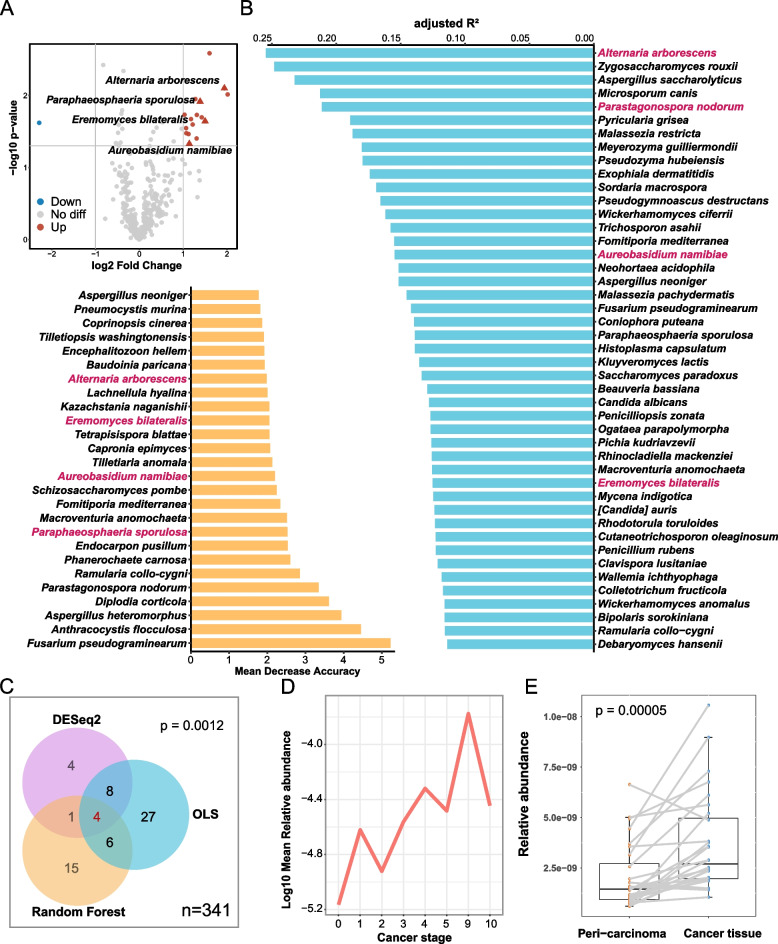
Detection of the relative key fungi with NSCLC. (**A**) Two methods are used to find key differential fungi between two groups. The volcano plot demonstrates the differential abundance of fungi between NSCLC and Non-NSCLC. Points are colored according to the number of log2 fold change with passed thresholds (*p* < 0.05 and |log2 fold change|> 1) which are calculated by DEseq2. The bar chart of key fungi between two groups which is determined by Random forest model (*p* < 0.05). (**B**) The fungi associated with NSCLC progress. We sorted each fungus by the adjusted R^2^ calculated from OLS model. Only the fungi with *p* < 0.05 and FDR < 0.2 are showed. (**C**) A venn graph shows the comprehensive results of the three methods. The hypergeometric test is used to check if the five key fungi detected by two methods (DEseq2 and Random Forest) enrich with the NSCLC development. (**D**) A line chart shows the variation between the log_10_ mean relative abundance of the *A. arborescens* and cancer stage. (**E**) Another cohort is used to confirm the enrichment of *A. arborescens* in the tissue of NSCLC (Paired Wilcoxon test, *p* = 0.00005)

To detect key fungi between the patients with NSCLC and the non-NSCLC, we used two different methods to reduce the randomness and inaccuracy of the algorithm. 17 and 20 distinct kinds of fungi were screened out by DESeq2 and Random Forest algorithm, respectively. After combining the two results, 5 kinds of fungi (*A. arborescens*, *Eremoyces bilateralis*, *Aureobasidium namibiae*, *Tilletiopsis washingtonensis* and *Paraphaeosphaeria sporulosa*) were detected as key fungi (Fig. 4A). In addition, the OLS model was used to predict the effect of correlation between the single fungus and NSCLC development. There were 45 kinds of fungi closely relative to NSCLC development (Fig. 4B), and these key fungi were significantly enriched with the NSCLC development (Fig. 4C*, p* = 0.0012). Not only was* A. arborescens* as the key fungus obtained by both algorithms, but also as the fungus with largest adjusted R^2^ to the progress of NSCLC in our OLS model. It was detected that its relative abundance was gradually increasing as NSCLC progressed (Fig. 4D). Although other three key fungi showed relationship (Additional file 1: Fig. S5B-D), none of them performed a great gradual upward trend. To test the generality of the correlation between *A. arborescens* and NSCLC, we performed the nested PCR on another cohorts (*n* = 28), and the results also showed *A. arborescens* significantly enriched in cancer tissues compared to peri-carcinoma tissues (Fig. 4E, *p* = 0.00005).

## Discussion

In this study, the higher fungal diversity and more complex network were observed in patients with NSCLC compared to Non-NSCLC group. Machine learning model based on the fungi relative abundance and cancer progress was built, and we found the alteration of fungi community relative to the NSCLC. In addition, *A. arborescens* was detected as the most relative fungus with NSCLC, and also showed significantly higher relative abundance in cancer tissues compared to that of para-cancerous tissues. These data suggest an association between a distinct human mycobiome and cancer in the lung.

Firstly, we provided the description of lung mycobiota composition, and the top two abundant kinds of fungi are *L. theobromae* and *M. globosa* in both groups. Although* L. theobromae* is a fungus as yet undescribed as a common resident of the lung microbiome, there are many fungi that do not have apparent host-specificity and are rather ubiquitous [31, 32]. Moreover, it is reported *L. theobromae* is associated with human health [33, 34]. *M. globosa* is also a skin-associated microbe and it was found relative to cancer in the recent studies [35, 36]. Secondly, the increased diversity of mycobiota was observed in the lung environment of patients with NSCLC, and the alpha-diversity of mycobiota in NSCLC patients was positively associated with clinical stages. There is a strong link between lung cancer, microbes, and inflammatory status [36–39]. Although the higher bacterial diversity was usually considered as benefits in the gut [40], the diversity of fungi increases significantly after infection [20]. This increased diversity was also detected in patients with Crohn’s disease [37, 41].

Co-occurrence network offers an approach to explore the microbial community structure, maintenance and dynamics [7], which can be applied to statistically explore the taxa that are highly connected in the community [42, 43]. As shown in the Fig. 2, the co-occurrence network analysis of fungi in this study suggested the variation in fungal communities and the enhanced complexity of interactions among fungi in patients with NSCLC. Meanwhile, in the UMAP analysis, most of the fungi which were added to the NSCLC network in red color were clustered around these two isolated blue nodes. This is consistent with the results in our extracted sub-networks analysis in Fig. 3. Compared to *S. octosporu*, there are more exclusive taxa in the NSCLC co-occurrence network occurred, when *T. asahii* was isolated from the Non-NSCLC network. *F. pseudograminearum* and *M. globosa* are not only normally detected in the environment but also detected in BAL samples [44]. In our study, they are also interactive nodes linked to altered nodes between two groups. It reported that the common microbial composition of lungs contains environment-microorganism and the composition of respiratory microbiota is influenced by environmental factors [45, 46].

Furthermore, the construction of machine learning-based regression models helps us to better understand the impact of different fungi and changing co-occurrence networks on the progression of NSCLC. As one of the key fungi, *A. arborescens* had the most significant correlation with the progression of NSCLC. Although *A. arborescens* mainly cause disease in plants, the airborne spores they produce can invade human respiratory tracts and cause respiratory and lung diseases.* A. arborescens* is one species of *Alternaria* genus. *Alternaria* is one kind of fungi that can also lead to infection in humans, and its spores are one of the most effective air allergens [47–50]. In addition, *Alternaria* is an opportunistic fungus, which can infect immunocompromised patients [51]. A recent study revealed that *Alternaria* can influence the development of cancer by affecting the immune system [36, 52]. Notably, several papers have reported the enrichment of microbes in cancer tissues and their possible role in the development of cancer by affecting immune and infection status [53–55]. In our study, another batch of samples from tumor and paracancerous tissues was also used to detect *A. arborescens* enrichment in cancerous tissues of NSCLC, and this enrichment increased credibility and avoided the bias of the results due to fungal infection. The reasons of alteration of lung microbiome can be varied and complex. This change can be mainly caused by microbial migration, elimination and growth rates [56–58]. It has also been reported that microbial metabolites of the gut may cause the immune system alterations thus affecting the respiratory microbiota [11, 59, 60]. Although microbes might directly affect protumorigenic pathways in epithelial cells [7], it is still unclear to determine the causal roles of fungal alterations.

Nevertheless, there are some limitations in our study. For instance, larger clinical cohorts need to be covered in the future. In addition, some microbial alterations in the lung caused by other conditions may compromise the results. Therefore, functional studies on mice are also needed to confirm our proposed effect of altered fungal community on NSCLC. Overall, our present study has taken the steps toward bringing a new perspective to elucidate the potential relationship between NSCLC and mycobiota. Based on detected fungal signatures, novel targeted treatment modalities in personalized medicine may emerge with the modification or restoration of a healthy fungal community in patients with cancer.

## Conclusions

In this study, we focused on the alteration of lung mycobiome in patients with NSCLC. Compared to Non-NSCLC group, the mycobiome in the lungs of patients with NSCLC showed greater fungal diversity. By using ridge regression model, we indicated the fungal co-occurrence network structure may be associated with NSCLC progress. In addition, *A. arborescens* was detected as the most relative key fungus to NSCLC, and we also found it enriched in the cancer tissues. Our study provides invaluable insights into further exploration in the relationship between NSCLC and fungi.

## Supplementary Information

**Additional file 1**: **Fig. S1** This is the scheme from sample processing to nested PCR. Firstly, the DNA of the tissue samples, Fungus positive control and the environment blank control (EBC) are extracted. Next, we performed the first amplification by PCR. And after that, the Agarose gel electrophoresis demonstrates contamination-free amplification. Lane 1: DNA marker, Lane 2: The 18S rRNA fragement DNA of *Malassezia globosa*, which is the positive control.  Lane 3 - 8: Six clinical samples were randomly selected for testing. Lane 9: The DNA of PBS, which is the negative control. In addition, 18S fragments including EBC (located at 300bp) are cut with UV light and extracted DNA again. At last, we performed the nested PCR to estimate the relative abundance of microbes. In this step, we additionally included the Non-template control group (NTC) used to exclude nested PCR reagent contamination. **Fig. S2** It shows the amplification and melting curves of the nested PCR. The graphs from top row represent the amplification curve of clinical samples and negative control, respectively. As for graphs from bottom row, they are melting curves. The two peaks in the left column represent the carcinoma tissue and the para-carcinoma group. There is no effective peak on the right column which means Negative control (EBC and NTC group). **Fig. S3** (A) barplot shows the fungal composition of both NSCLC and Non-NSCLC groups. The top 15 fungi in terms of relative abundance were shown in the picture, and other were classified as ‘Others’. (B) Species rarefaction curves in red and black indicate NSCLC and non-NSCLC groups, respectively. (C) Differences in beta-diversity between the mycobiome in patients with NSCLC and non-NSCLC groups were estimated based on a Bray-Curtisdistance matrix of all 38 samples (Wilcoxon test, *p* = 0.003). (D) The fungal content of patients with NSCLC and Non-NSCLC groups by using qPCR (t test, *p* =0.031). **Fig. S4** The degree distribution for co-occurrence networks in NSCLC and Non-NSCLC group, respectively. The *p*-values are calculated by using permutation test. **Fig. S5** (A) The plot only shows the fungi of which degree is not zero in NSCLC co-occurrence network, and different points mean different fungi’s adjusted R^2^. Based on their distribution, we fit these points to a trend line, and calculated *p* value and R^2^.The *p*-values are calculated by permutation test. (B, C and D) The line charts show the variation between the log_10_ mean relative abundance of different fungi and cancer stage.

## Data Availability

The raw sequence data reported in this paper have been deposited in the Genome Sequence Archive in National Genomics Data Center, China National Center for Bioinformation/Beijing Institute of Genomics, Chinese Academy of Sciences, under accession number CRA006566, which publicly accessible at https://bigd.big.ac.cn/gsa.

## Abbreviations

NSCLC: Non-small-cell lung cancer
BAL: Bronchoalveolar lavage
OLS: Ordinary Least Square
HMP: Human Microbiome Project
PCoA: Principal co-ordinates analysis
PERMANOVA: Permutational multivariate analysis of variance
UMAP: Uniform Manifold Approximation and Projection
