# Supplementary material for: Exploration of lung mycobiome in the patients with non-small-cell lung cancer
Source: BMC Microbiol. 2023 Mar 25;23:81. doi: 10.1186/s12866-023-02790-4 (PMC10039514; doi:10.1186/s12866-023-02790-4)
Supplement: Supplementary file 1 — Additional file 1: Fig. S1 This is the scheme from sample processing to nested PCR. Firstly, the DNA of the tissue samples, Fungus positive control and the environment blank control (EBC) are extracted. Next, we performed the first amplification by PCR. And after that, the Agarose gel electrophoresis demonstrates contamination-free amplification. Lane 1: DNA marker, Lane 2: The 18S rRNA fragement DNA of Malassezia globosa, which is the positive control. Lane 3 - 8: Six clinical samples were randomly selected for testing. Lane 9: The DNA of PBS, which is the negative control. In addition, 18S fragments including EBC (located at 300bp) are cut with UV light and extracted DNA again. At last, we performed the nested PCR to estimate the relative abundance of microbes. In this step, we additionally included the Non-template control group (NTC) used to exclude nested PCR reagent contamination. Fig. S2 It shows the amplification and melting curves of the nested PCR. The graphs from top row represent the amplification curve of clinical samples and negative control, respectively. As for graphs from bottom row, they are melting curves. The two peaks in the left column represent the carcinoma tissue and the para-carcinoma group. There is no effective peak on the right column which means Negative control (EBC and NTC group). Fig. S3 (A) barplot shows the fungal composition of both NSCLC and Non-NSCLC groups. The top 15 fungi in terms of relative abundance were shown in the picture, and other were classified as ‘Others’. (B) Species rarefaction curves in red and black indicate NSCLC and non-NSCLC groups, respectively. (C) Differences in beta-diversity between the mycobiome in patients with NSCLC and non-NSCLC groups were estimated based on a Bray-Curtisdistance matrix of all 38 samples (Wilcoxon test, p = 0.003). (D) The fungal content of patients with NSCLC and Non-NSCLC groups by using qPCR (t test, p =0.031). Fig. S4 The degree distribution for co-occurrence networks in NSCLC [file 12866_2023_2790_MOESM1_ESM.docx]

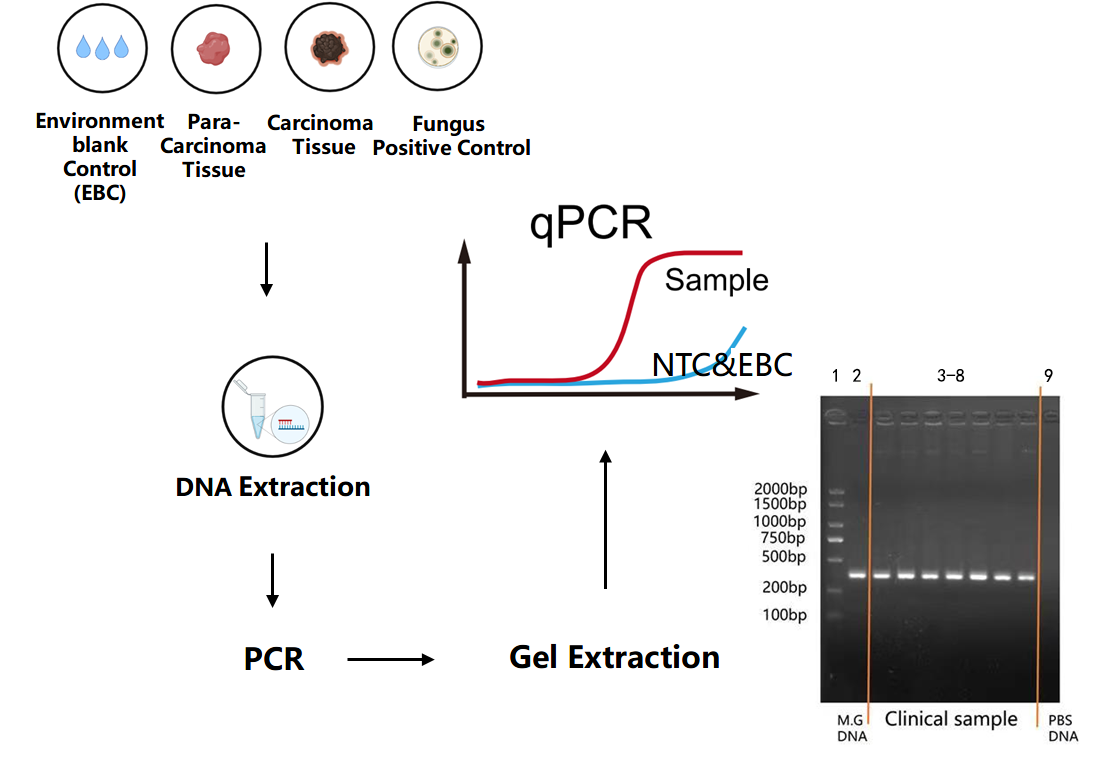


**Fig. S1** This is the scheme from sample processing to nested PCR. Firstly, the DNA of the tissue samples, Fungus positive control and the environment blank control (EBC) are extracted. Next, we performed the first amplification by PCR. And after that, the Agarose gel electrophoresis demonstrates contamination-free amplification. Lane 1: DNA marker, Lane 2: The 18S rRNA fragement DNA of *Malassezia globosa*, which is the positive control. Lane 3 - 8: Six clinical samples were randomly selected for testing. Lane 9: The DNA of PBS, which is the negative control. In addition, 18S fragments including EBC (located at 300bp) are cut with UV light and extracted DNA again. At last, we performed the nested PCR to estimate the relative abundance of microbes. In this step, we additionally included the Non-template control group (NTC) used to exclude nested PCR reagent contamination.


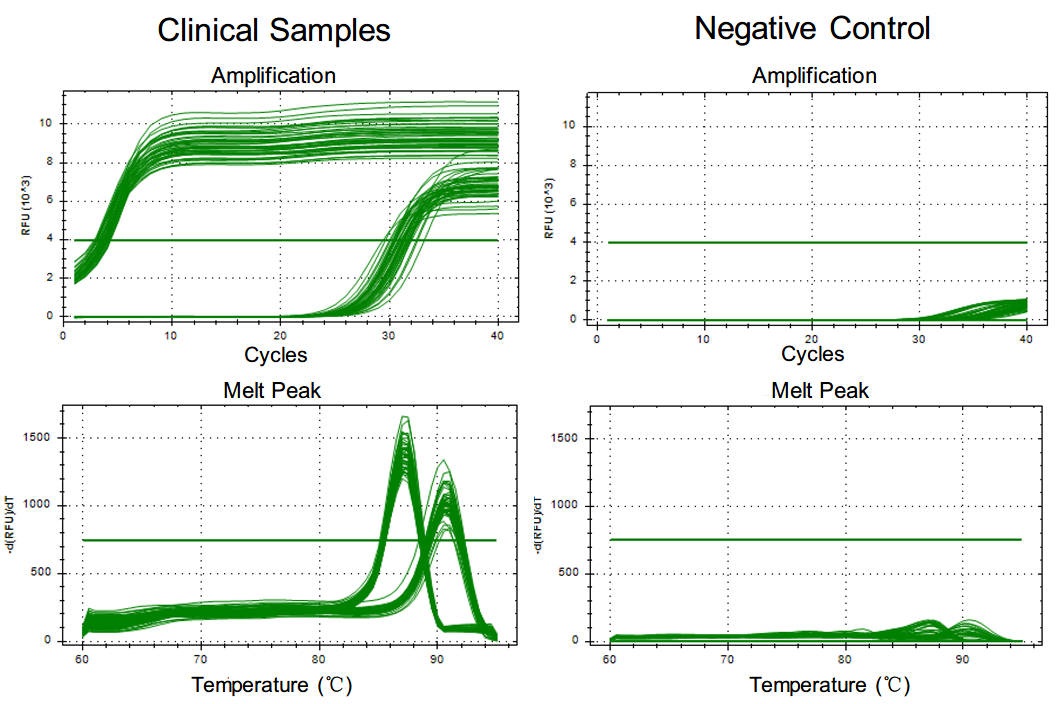


**Fig. S2** It shows the amplification and melting curves of the nested PCR. The graphs from top row represent the amplification curve of clinical samples and negative control, respectively. As for graphs from bottom row, they are melting curves. The two peaks in the left column represent the carcinoma tissue and the para-carcinoma group. There is no effective peak on the right column which means Negative control (EBC and NTC group).


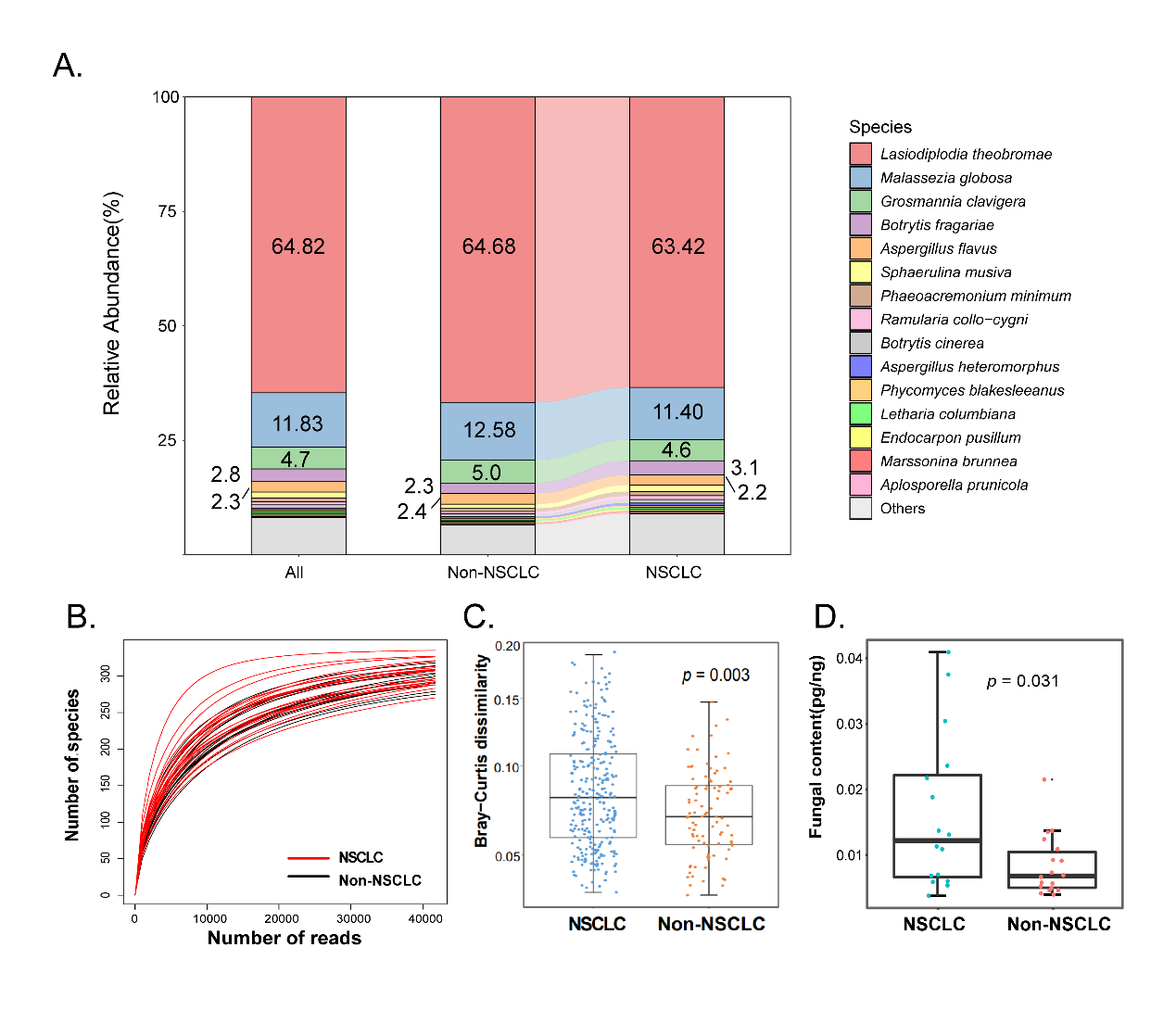


**Fig. S3** (A) barplot shows the fungal composition of both NSCLC and Non-NSCLC groups. The top 15 fungi in terms of relative abundance were shown in the picture, and other were classified as ‘Others’. (B) Species rarefaction curves in red and black indicate NSCLC and non-NSCLC groups, respectively. (C) Differences in beta-diversity between the mycobiome in patients with NSCLC and non-NSCLC groups were estimated based on a Bray-Curtis distance matrix of all 38 samples (Wilcoxon test, *p* = 0.003). (D) The fungal content of patients with NSCLC and Non-NSCLC groups by using qPCR (t test, *p* = 0.031).


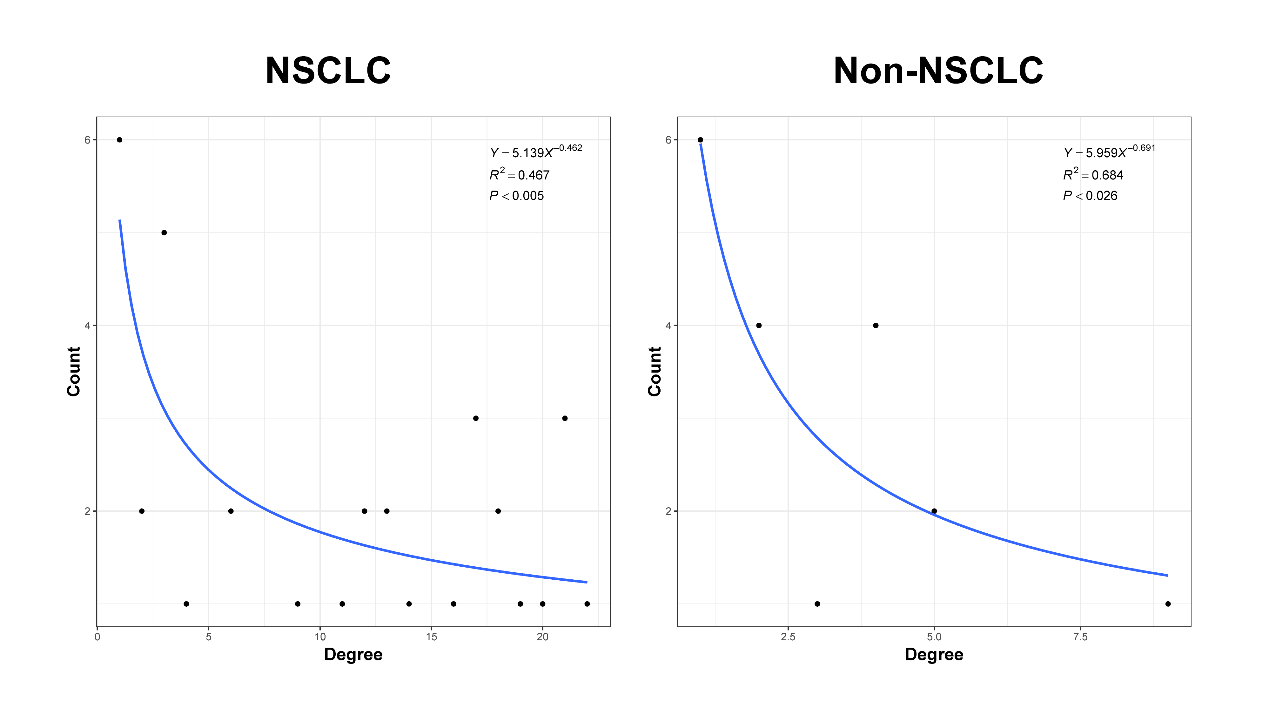


**Fig. S4** The degree distribution for co-occurrence networks in NSCLC and Non-NSCLC group, respectively. The p-values are calculated by using permutation test.

**
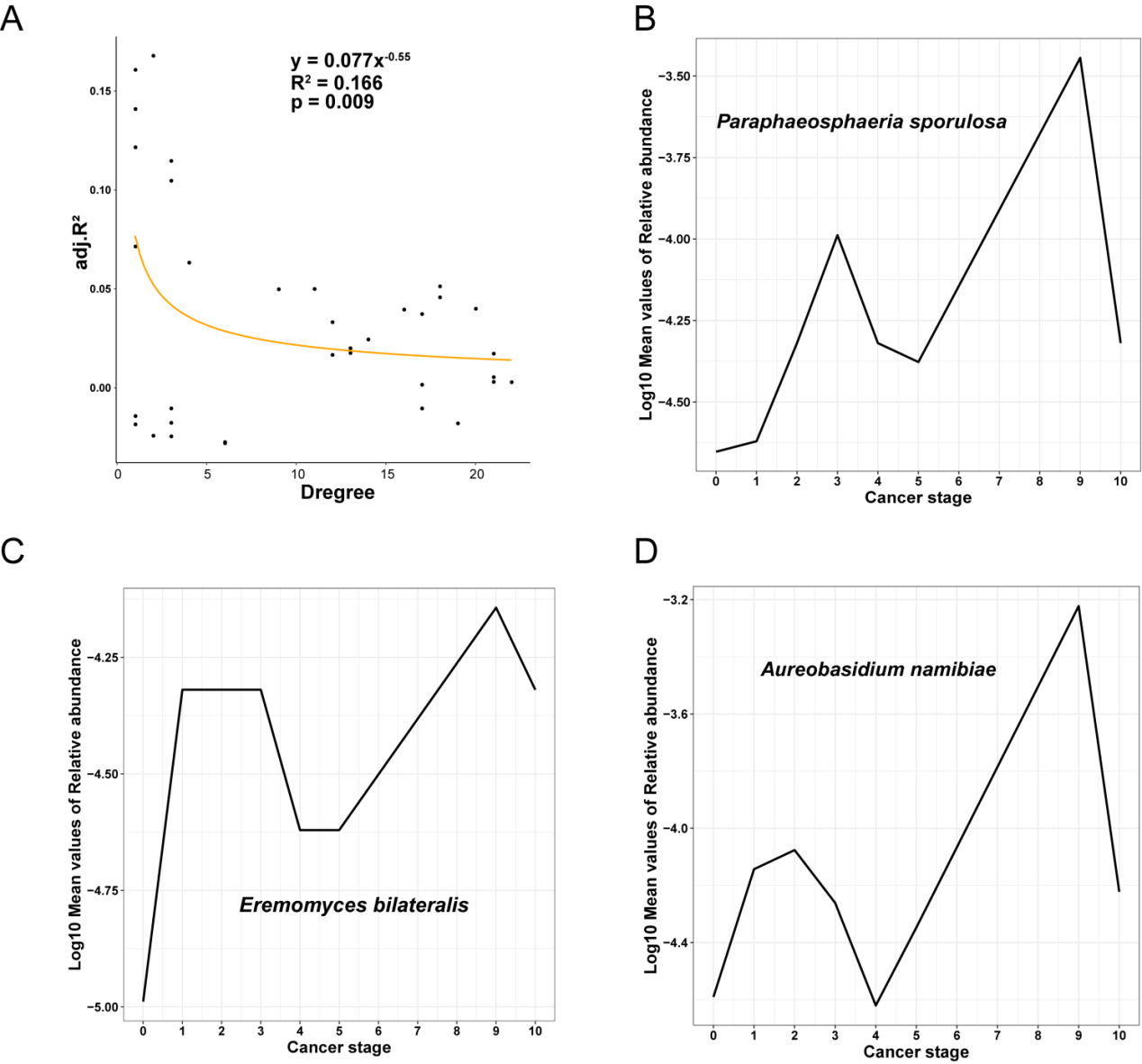
**

**Fig. S5** (A) The plot only shows the fungi of which degree is not zero in NSCLC co-occurrence network, and different points mean different fungi’s adjusted R^2^. Based on their distribution, we fit these points to a trend line, and calculated p value and R^2^. The p-values are calculated by permutation test. (B, C and D) The line charts show the variation between the log_10_ mean relative abundance of different fungi and cancer stage.
